# Study on the regional risk classification method for the prevention and control of emerging infectious diseases based on directed graph theory

**DOI:** 10.3389/fpubh.2023.1211291

**Published:** 2023-09-25

**Authors:** Yong Liu, Xiao Wang, Chongqi Zhang

**Affiliations:** ^1^School of Science, Xi'an University of Architecture and Technology, Xi'an, China; ^2^School of Economics and Statistics, Guangzhou University, Guangzhou, China

**Keywords:** scale-free network, emerging infectious diseases, graph theory, grading and zoning, epidemic

## Abstract

**Background:**

Emerging infectious diseases are a class of diseases that are spreading rapidly and are highly contagious. It seriously affects social stability and poses a significant threat to human health, requiring urgent measures to deal with them. Its outbreak will very easily lead to the large-scale spread of the virus, causing social problems such as work stoppages and traffic control, thereby causing social panic and psychological unrest, affecting human activities and social stability, and even endangering lives. It is essential to prevent and control the spread of infectious diseases effectively.

**Purpose:**

We aim to propose an effective method to classify the risk level of a new epidemic region by using graph theory and risk classification methods to provide a theoretical reference for the comprehensive evaluation and determination of epidemic prevention and control, as well as risk level classification.

**Methods:**

Using the graph theory method, we first define the network structure of social groups and construct the risk transmission network of the new epidemic region. Then, combined with the risk classification method, the classification of high, medium, and low risk levels of the new epidemic region is discussed from two cases with common and looped graph nodes, respectively. Finally, the reasonableness of the classification method is verified by simulation data.

**Results:**

The directed weighted scale-free network can better describe the transmission law of an epidemic. Moreover, the proposed method of classifying the risk level of a region by using the correlation function between two regions and the risk value of the regional nodes can effectively evaluate the risk level of different regions in the new epidemic region. The experiments show that the number of medium and high risk nodes shows no increasing trend. The number of high-risk regions is relatively small compared to medium-risk regions, and the number of low-risk regions is the largest.

**Conclusions:**

It is necessary to distinguish scientifically between the risk level of the epidemic area and the neighboring regions so that the constructed social network model of the epidemic region's spread risk can better describe the spread of the epidemic risk in the social network relations.

## 1. Introduction

Emerging infectious diseases (EIDs) are a class of diseases with a wide range of transmission, multiple modes of transmission, incidence rates much higher than the annual incidence level, difficulty of control, ease of infection of the population, lack of specific treatment and prevention methods (1) and other characteristics. Once an EIDs occurs, it will spread faster and cover a wider area than traditional infectious diseases, causing social problems, affecting the global economy, and even endangering human lives. However, we note that the risk of an epidemic varies from region to region, and people's concerns and social sentiments in different regions also differ. Therefore, a practical classification of the risk level of an epidemic area and the implementation of targeted prevention and control measures according to the risk level are effective means of controlling the rapid spread of the epidemic.

There is a wealth of literature on analyzing the transmission patterns of infectious disease outbreaks and preventative and control measures from various perspectives. On the one hand, studies focus on analyzing and grasping the causes of the occurrence and spread of emerging infectious diseases. For example, Lashley (2) analyzed the factors influencing the occurrence of common emerging infectious diseases (EIDs) and concluded that microbial characteristics are essential in the emergence of infectious diseases. However, humans' behaviors and lifestyle choices are major factors in the emergence and spread of many EIDs. Sabin et al. (3) show how several factors related to human activities play a role in spreading infectious diseases and discuss the main factors contributing to the global spread of the COVID-19 pandemic. Yang and Zhang (4) summarized the uncertainty and complexity of EID and provided the preventive measures for dealing with EIDs.

On the other hand, the model is an effective mathematical tool to analyze and predict infectious disease transmission modes and laws. For example, Chu et al. (5) studied the epidemic spreading in weighted scale-free networks with community structure based on the SI disease model and showed the hierarchical dynamics of the epidemic spreading in the weighted scale-free networks with communities. Sun et al. (6) introduced three modified SIS models on scale-free networks that take into account variable population size, non-linear infectivity, adaptive weights, behavior inertia, and time delay, which could better characterize the actual spread of epidemics. Li et al. (7) using China's prefecture-level high-speed rail network and based on a probabilistic risk model, assessed the risk of COVID-19 infection in 19 provincial-level regions from Wuhan to the whole country in the early stage of domestic transmission, and found that the probability and impact could play different roles in the risk ranking of different regions. Shi et al. (8) developed a comprehensive model for simulating and predicting emerging infectious diseases, based on transmission dynamics and a statistical model driven by public health data. Chowell et al. (9) used the early exponential growth rate method to propose a simple susceptibility-exposure-infection-recovery (SEIR) model, a more complex SEIR model with asymptomatic and hospitalized cases and a stochastic susceptibility-infection-removal (SIR) model with Bayesian estimation to estimate the reproduction number of Spanish influenza, respectively. Bentout et al. (10) predict the peak time and the number of infectious cases at the peak before and after the implementation of non-pharmaceutical interventions for the COVID-19 based on an age-structured model. Reema et al. (11) compares the effectiveness of the SIR and SEIR models in analyzing epidemic data and also discusses how measures such as social distance and vaccination affect virus transmission in the SIR model.

Mathematical models based on differential equations provide statistical results consistent with the situation in practice, which is an effective tool for studying EIDs. However, as mentioned by Yu and Xue (12), most of the models based on differential dynamical systems are generally computationally cumbersome, and the solutions of the equations are extremely sensitive to the initial conditions, so they cannot deal with the unexpected and random events in the actual process well. In addition, the system formed by the communication, contact, and linkage between people is complex, which leads to the complexity of the transmission process of infectious diseases.

Graph theory is an ideal tool for analyzing, modeling, predicting and forming opinions to formulate strategies to rapidly contain the epidemic and minimize the devastating effects of viral infections (13). Baagyere et al. (14) characterized several complex networks from different domains using concepts from graph theory, and the node degrees, graph spectral radius, degree assortativity, and the entire topological structure of selected complex networks are studied on the SIR epidemic model. It is a fact that graph theory and complex networks are inevitably related. For a complex network, if not considering its dynamic features, the complex network is a graph, and the relevant features of the graph, such as subgraph and complementary graph features, have great significance for the modeling of complex networks (15). In addition, the classical infectious disease model combined with the complex network structure is important for investigating and analyzing the EIDs. Note that a social network (16, 17) is a complex network system based on the relationship between people and established according to certain rules. Zhang et al. (18) constructed an interpersonal network model, and the experimental results indicate that it is feasible and valuable to study virtual social simulation. Moore and Newman (19) studied the transmission characteristics of infectious diseases through the small-world network model and found that the nature of the small world would accelerate the transmission of infectious diseases. Huang et al. (20) found that the scale-free network nodes (21–25) in the complex network satisfy the power law distribution, which is more consistent with the real social network and the transmission law of infectious diseases in the entire society.

In addition, the effective division of regional risk levels is significant for preventing and controlling the rapid spread of EIDs. At present, relevant research has focused on studying regional risk classification methods. For example, Jia et al. (26) constructed an epidemic risk assessment model based on the analysis of population flow data and evaluated the degree of risk for each city using the collected population flow data related to Wuhan, China. Li et al. (27) evaluated the risk level of 38 districts in Chongqing, China, using the single index evaluation method, the analytical hierarchy process, and the systematic clustering method, respectively. Based on the population migration during COVID-19, Feng et al. (28) constructed a migratory imported risk index by using the number of accumulated cases and the number of new cases and showed that the index could better evaluate the epidemic risk in different places. Tu et al. (29) designed the scoring system with expert consultation and calculated the import, spread, and combined risk scores of regions using quantitative analysis methods to determine the risk level. Using unsupervised machine learning techniques, Fidan et al. (30) applied two clustering methods to classify COVID-19 risk degree.

In this paper, we consider the whole epidemic area as a scale-free network and classify the risk level for each area according to the association between regions. Meanwhile, combining the complex networks with risk assessment models to analyze the risk transmission of infectious diseases process and provide a theoretical basis for evaluating the prevention and control of epidemics and the risk classification of the region. The paper is organized as follows. Section 2 presents the steps for constructing social networks in new epidemic regions. Section 3 presents the method for building the epidemic classification model. Section 4 Simulation of the effectiveness of the method. Section 5 concludes with a brief discussion.

## 2. Construction of the directed weighted scale-free networks in new epidemic regions

Since infectious disease transmission has been oriented, the directed weighted scale-free networks (31) are more suitable to describe the spread of epidemic risk in real social networks. In the following, we introduce the method of defining the social network as scale-free one and provide the main step for constructing the directed weighted scale-free networks in new epidemic regions.

Let *E*⊆*V*×*V* be the edge set, as introduced by Pastor-Satorras et al. (32), if *V* = {1, 2, ⋯ , *N*} is the node set, then the directed weighted network constructed by *N* nodes is denoted as *G* = (*V, E*).

Now, we introduce the necessary notions as follows. Let *k*_*m*_ be the number of edges connected to the node *m*. It can be divided into in-degree $k_{m}^{in}$ and out-degree $k_{m}^{out}$ for a directed graph, where $k_{m}^{in}$ is the number of directed edges ending at node *m* and $k_{m}^{out}$ is the number of directed edges starting from node *m*.

In addition, suppose that *s*_*m*_ is the strength of node *m*, and it can be divided into vertex in weight $s_{m}^{in}$ and vertex out weight $s_{m}^{out}$ for a directed graph, where $s_{m}^{in}$ is the sum of the weights of all edges reaching node *m*, $s_{m}^{out}$ is the sum of the weights of all edges starting from node *m*.

Moreover, let *w*_*m, s*_ be the weight of the connection between the node *m* and *s*, then the weight of a weighted network is divided into edge weight *w*_*m, s*_ and point weight *w*_*s, m*_, and the *w*_*m, s*_≠*w*_*s, m*_, *w*_*m, s*_≠ 0 if *m*→*s* are connected.

In the following, under the necessary notions above, we will introduce the main steps of the directed weighted scale-free network construction method provided by Barabási et al. (33).

**Step 1** *Starting from a network with only two nodes, a new node is added each time and connected to existing ones*.

**Step 2** *Assuming that the node* *m* *is an existing node, the node with the higher degree is preferentially connected when a new node is added. Suppose that the node* *s* *is a new node, the probability of node* *s* *connecting to node* *m* *is*


$$\begin{array}{l} P\left(k_{s}\right)=\frac{k_{s}}{\underset{m}{\sum }k_{m}}, \end{array}$$

where *k*_*s*_ and *k*_*m*_ represent the degree of node *s* and *m*, respectively.

**Step 3** *Repeat steps 1 and 2 above until the target number of points and edges is reached and the directed weighted scale-free network is constructed*.

## 3. Method for determining the risk level of epidemic regions

In this section, we will present the methodology for determining the risk level of the epidemic regions.

Now, we assume that the division of regional units is consistent and relatively independent. Let *v*_*m*_ be a node of a new epidemic region. The edge of the network represents the social connection between the two regions, and the weight of the edge represents the correlation between the two regions *m* and *s*, usually represented by the correlation function denoted by *L*_*m, s*_. Note that an outbreak in one region will affect all connected regions, and the size of *L*_*m, s*_ directly determines the size of the epidemic in the region.

### 3.1. Determination of risk correlation function

To determine the correlation strength *L*_*m, s*_ between two regions, we will choose *t* indicators that affect the *L*_*m, s*_ based on the security principle of each region and the relevant personnel and the minimum impact on the economy. In this paper, the five indicators chosen are the distance between two regions, personnel flow, economic traffic, transport convenience, and logistics intensity.

Here we consider the importance of the criteria through the intercriteria correlation (*CRITIC*) method (34) to determine the weight of the indicators *t* and the correlation function *L*_*m, s*_ between the two regions *m* and *s*, which the *CRITIC* measures the objective weight of the indicators based on the comparative strength of the evaluation indicators and the conflict between indicators, and the main steps of the method as follows:

**Step 1** *Assuming that there are* *h* *regions of interest and* *t* *indicators that influence the correlation strength, let the matrix consisting of the data of the* *i*(*i* = 1, 2, ⋯ , *t*)*th indicator be*


$$B_{i}=\left[\begin{array}{cccc} b_{i(11)} & b_{i(12)} & \cdots & b_{i(1h)}\\ b_{i(21)} & b_{i(22)} & \cdots & b_{i(2h)}\\ \vdots & \vdots & \vdots & \vdots \\ b_{i(h1)} & b_{i(h2)} & \cdots & b_{i(hh)} \end{array}\right],$$

where *b*_*i*(*ms*)_ is the original data of the indicator *i* between regions *m* and *s*.

**Step 2** *Denote*


$$\begin{array}{l} S_{i}=\left[\begin{array}{cccc} s_{i(11)} & s_{i(12)} & \cdots & s_{i(1h)}\\ s_{i(21)} & s_{i(22)} & \cdots & s_{i(2h)}\\ \vdots & \vdots & \vdots & \vdots \\ s_{i(h1)} & s_{i(h2)} & \cdots & s_{i(hh)} \end{array}\right], \end{array}$$

where *s*_*i*(*ms*)_ is the index value of *b*_*i*(*ms*)_ by dimensionless (35). If the size of *b*_*i*(*ms*)_ is proportional to the risk, we have


$$s_{i\left(ms\right)}=\frac{b_{i\left(ms\right)}-\min\left(b_{i\left(ms\right)}\right)}{\max\left(b_{i\left(ms\right)}\right)-\min\left(b_{i\left(ms\right)}\right)},$$

otherwise,


$$s_{i\left(ms\right)}=\frac{\max\left(b_{i\left(ms\right)}\right)-b_{i\left(ms\right)}}{\max\left(b_{i\left(ms\right)}\right)-\min\left(b_{i\left(ms\right)}\right)}.$$

**Step 3** *Let*


$$\bar{s_{i\left(ms\right)}}=\frac{1}{hh\overset{hh}{\underset{s=1}{\sum }}s_{i\left(ms\right)}}.$$

*Then, the standard deviation of the* *i-th index is given by*


$$P_{i}=\sqrt{\frac{{\overset{hh}{\underset{s=1}{\sum }}\left(s_{i\left(ms\right)}-\bar{s_{i\left(ms\right)}}\right)}^{2}}{hh-1}}.$$

**Step 4** *Let* *R*_*i*_ *be a measure of the conflict created by the* *j-th indicator with respect to the* *i-th indicator, we have*


(1) $$\begin{array}{l} R_{i}=\overset{t}{\underset{j=1}{\sum }}\left(1-r_{ij}\right), \end{array}$$

where *r*_*ij*_ represents the correlation coefficient between the evaluation index *i* and *j*.

**Step 5** *Denote*


$$C_{i}=P_{i}\overset{t}{\underset{j=1}{\sum }}\left(1-r_{ij}\right)=P_{i}R_{i}.$$

*Then, the objective weight is defined as*


$$w_{i}=\frac{C_{i}}{\overset{t}{\underset{i=1}{\sum }}C_{i}}.$$

**Step 6** *The weighted standardized matrix* *R* = (_*r*_*i*(*ms*)_)*t*(*hh*)_ *is obtained, where* *r*_*i*(*ms*)_ = *w*_*i*_*s*_*i*(*ms*)_, *w*_*i*_ *is the index weight determined by the above steps*.

Following the above steps, the correlation function *L*_*m, s*_ between the two regions *m* and *s* is


(2) $$\begin{array}{l} L_{m,s}=\overset{t}{\underset{i=1}{\sum }}w_{i}*s_{i\left(ms\right)} \end{array}$$

### 3.2. Calculation of node risk function and risk value

Let *p* be the value of the risk of the epidemic occurring in a region of the social network. If the epidemic occurs in this region, *p* = 1, while for other regions (nodes), *p* = 0. We first define the nodes that are connected to the nodes in layer 1 (in addition to the defined nodes) as layer 2 nodes, and so on until we have defined all the nodes in the network. Note that if an infectious disease occurs in a node (region), another node directly connected to that node will be the first affected node, i.e., a layer 1 node. In particular, if the node is the first node in the scale-free network, the risk value is 1.

Furthermore, we define the direction of the edges as the direction of the epidemic spread and construct a weighted scale-free network for the spread of risk across the epidemic region. Since scale-free networks have cyclic graphs (36) and are calculated differently from ordinary nodes. In the following, we focus on two different node cases to provide methods for computing the risk function and risk value, respectively.

**Case 1: Calculation of risk value of common node**

Now, starting from the node whose risk value is 1 (the initial node), we calculate the risk value of the first layer node connected to it. According to the direction of risk transmission, if the in-degree of node *s* is 1, it shows that node *s* is influenced by a node. Assuming that node *s* is influenced by node *m*, the risk value of node *s* can be defined as the product of the risk value of node *m* and the association function between two nodes as follows:


(3) $$\begin{array}{l} p_{s}=p_{m}L_{m,s}, \end{array}$$

where *p*_*s*_ is the risk value of the node *s* and *p*_*m*_ is the risk value of the node *m*.

Let the set π(*s*_*n*_) represents the set of all other nodes that affect the node s, then the risk value can be defined as the sum of the risk value and the correlation function product of the node s and all the nodes *s*_*n*_ on π(*s*_*n*_), that is 


(4) $$\begin{array}{l} p_{s}=\underset{s_{n}\in \pi_{\left(s_{n}\right)}}{\sum }p_{s_{n}}l_{s_{n},s}L_{s_{n},s}, \end{array}$$

where


$$\begin{array}{l} l_{s_{n},s}=\frac{L_{s_{n},s}}{\underset{s_{n}\in \pi_{\left(s_{n}\right)}}{\sum }L_{s_{n},s}}. \end{array}$$

Therefore, starting from each node where we have obtained the risk value, we find the nodes connected to it and then calculate the risk value of all nodes in the obtained scale-free network according to Equations (3) and (4).

**Case 2: Calculation of risk value of the loop graph node**

Note that the risk transmission direction of nodes at different levels is transferred from the upper node to the lower node, the risk transmission is unidirectional. If all the nodes are only influenced by their upper-layer nodes, the risk value of the node is only related to the upper-layer nodes, and the node does not influence the risk value of the upper-layer nodes. Therefore, the method of calculating the risk value of each node is the same as that of the common nodes, and the Equations (3) and (4) can be used directly to calculate the risk values of nodes in different layers. However, it should be noted that for a loop graph node there is no order between them that can influence each other, and the risk transmission direction is bidirectional when the nodes of the same layer are connected, and Equations (3) and (4) cannot be used.

#### 3.2.1. Classification of risk levels

Using the same method of the three-level risk rating system, as introduced in Tu et al. (37), we have the classification criteria as shown in Table 1.

**Table 1:** Risk classification criteria.

**Risk level**	**High-risk**	**Medium-risk**	**Low-risk**
Value at risk	*p* ≥ *p*_*s*_1__	*p*_*s*_2__ ≤ *p* < *p*_*s*_1__	*p* < *p*_*s*_2__

It follows that the risk classification criteria given in Table 1, we need to calculate the risk value of each node to accurately classify the risk level of the area represented by all nodes. Then, according to Table 1, the whole area can be classified into high risk, medium risk and low risk, and the corresponding precise prevention and control measures can be taken for the different risk level of each region.

## 4. Experiments and simulation

In this section, we aim to verify the feasibility of the regional risk classification method proposed in this paper by simulation. Firstly, we use *MATLAB* software to construct a scale-free network with nodes that follow a power law distribution and have the characteristics of a real network. Secondly, we consider five necessary indicators that influence the correlation intensity of regional risk, namely the distance between two regions, personnel flows, economic traffic, transport convenience and logistics intensity, and use the *MATLAB* software to generate two sets of original data for each of the above five indicators, as shown in the Supplementary material. Moreover, using Equation (2), we calculate the correlation intensity of two sets of original data for these five indicators as shown in Tables 2, 3 below. Finally, we analyzed the two sets of experimental data using the presented classification method of regional risk levels.

**Table 2:** The correlation function for group 1.

*L*_1, 2_ = 0.7117	*L*_1, 3_ = 0.3215	*L*_1, 4_ = 0.4431	*L*_1, 5_ = 0.6580		
*L*_2, 22_ = 0.8179	*L*_2, 23_ = 0.3893	*L*_2, 24_ = 0.2132	*L*_2, 25_ = 0.8065		
*L*_3, 16_ = 0.4333	*L*_3, 17_ = 0.8896	*L*_3, 18_ = 0.7086	*L*_3, 19_ = 0.0171	*L*_3, 20_ = 0.1932	*L*_3, 21_ = 0.8897
*L*_4, 6_ = 0.9380	*L*_4, 7_ = 0.6014				
*L*_5, 11_ = 0.1752	*L*_5, 12_ = 0.1096	*L*_5, 13_ = 0.7432	*L*_5, 14_ = 0.2404	*L*_5, 15_ = 0.6849	
*L*_8, 22_ = 0.8328	*L*_8, 47_ = 0.1397	*L*_8, 48_ = 0.7896	*L*_8, 49_ = 0.1932	*L*_8, 50_ = 0.4378	
*L*_9, 22_ = 0.7266					
*L*_10, 22_ = 0.3939					
*L*_13, 14_ = 0.9448					
*L*_23, 35_ = 0.7992	*L*_23, 36_ = 0.6164	*L*_23, 37_ = 0.2802	*L*_23, 38_ = 0.0652	*L*_23, 39_ = 0.8393	*L*_23, 40_ = 0.5297
*L*_24, 31_ = 0.9806	*L*_24, 32_ = 0.0496	*L*_24, 33_ = 0.9852	*L*_24, 34_ = 0.3359		
*L*_25, 26_ = 0.9701	*L*_25, 27_ = 0.8291	*L*_25, 28_ = 0.6448	*L*_25, 29_ = 0.2832	*L*_25, 30_ = 0.5597	
*L*_38, 41_ = 0.6088	*L*_38, 42_ = 0.0043	*L*_38, 43_ = 0.2152	*L*_38, 44_ = 0.5119	*L*_38, 45_ = 0.8964	*L*_38, 46_ = 0.6535
*L*_42, 43_ = 0.2537					
*L*_48, 49_ = 0.8281					

**Table 3:** The correlation function for group 2.

*L*_1, 2_ = 0.8147	*L*_1, 3_ = 0.2760	*L*_1, 4_ = 0.1622	*L*_1, 5_ = 0.4173		
*L*_2, 22_ = 0.6433	*L*_2, 23_ = 0.9361	*L*_2, 24_ = 0.0596	*L*_2, 25_ = 0.3015		
*L*_3, 16_ = 0.4299	*L*_3, 17_ = 0.9160	*L*_3, 18_ = 0.5822	*L*_3, 19_ = 0.7363	*L*_3, 20_ = 0.8507	*L*_3, 21_ =0.6797
*L*_4, 6_ = 0.7943	*L*_4, 7_ = 0.0479				
*L*_5, 11_ = 0.3789	*L*_5, 12_ = 0.5468	*L*_5, 13_ = 0.6802	*L*_5, 14_ = 0.7011	*L*_5, 15_ = 0.0942	
*L*_8, 22_ = 0.5407	*L*_8, 47_ = 0.3947	*L*_8, 48_ = 0.5606	*L*_8, 49_ = 0.9448	*L*_8, 50_ = 0.3112	
*L*_9, 22_ = 0.9027					
*L*_10, 22_ = 0.0714					
*L*_13, 14_ = 0.9448					
*L*_23, 35_ = 0.6433	*L*_23, 36_ = 0.0376	*L*_23, 37_ = 0.8116	*L*_23, 38_ = 0.6663	*L*_23, 39_ = 0.5985	*L*_23, 40_ = 0.4624
*L*_24, 31_ = 0.8699	*L*_24, 32_ = 0.6834	*L*_24, 33_ = 0.9296	*L*_24, 34_ = 0.9457		
*L*_25, 26_ = 0.5328	*L*_25, 27_ = 0.2316	*L*_25, 28_ = 0.0714	*L*_25, 29_ = 0.52391	*L*_25, 30_ = 0.4709	
*L*_38, 41_ = 0.4243	*L*_38, 42_ = 0.2684	*L*_38, 43_ = 0.7040	*L*_38, 44_ = 0.6967	*L*_38, 45_ = 0.3507	*L*_38, 46_ = 0.4889
*L*_42, 43_ = 0.0516					
*L*_48, 49_ = 0.6981					

### 4.1. Construction of a scale-free social network

**Figure 1:**
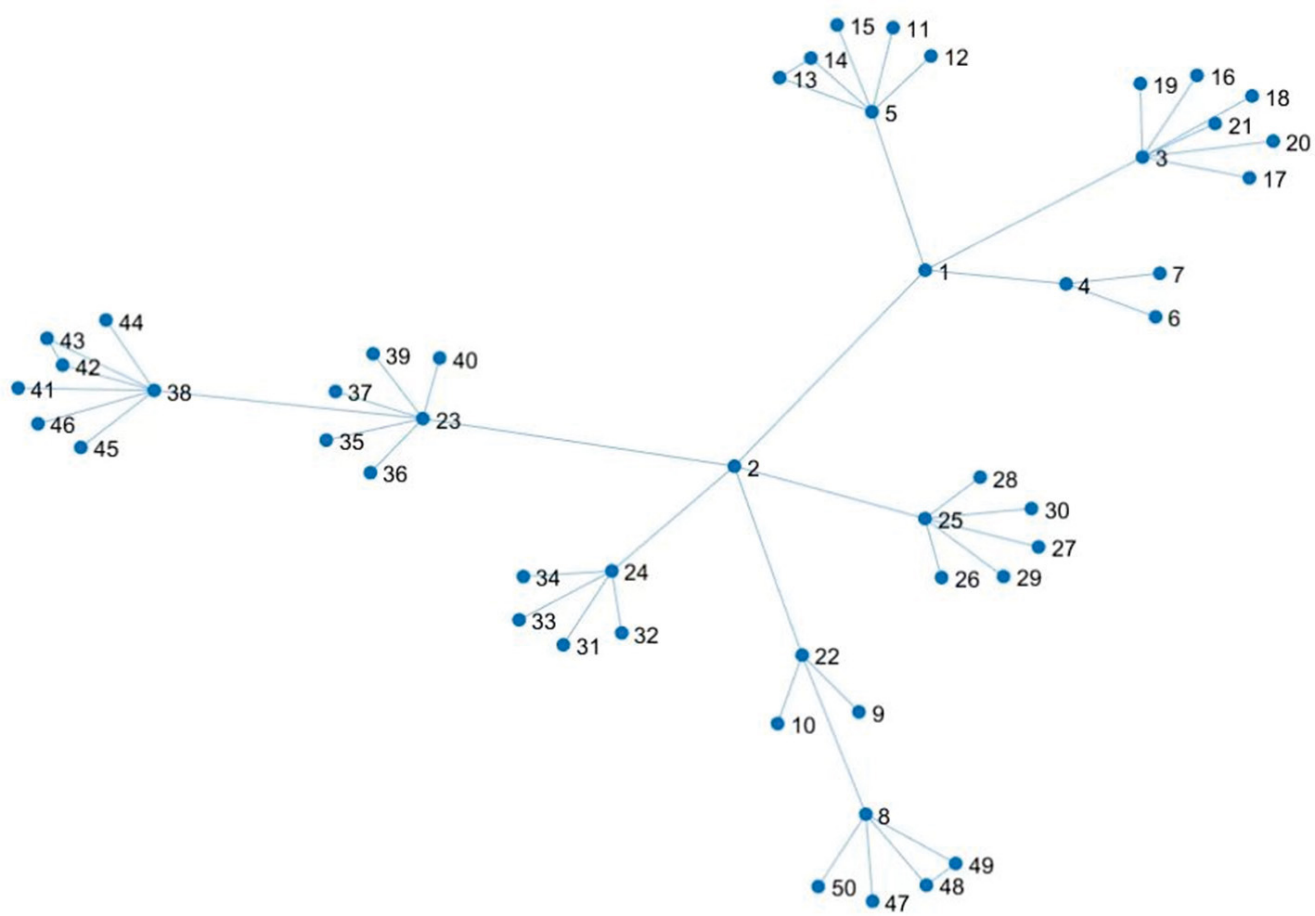
Scale-free social networks.

Using the *MATLAB* software, we construct the scale-free network as shown in Figure 1. There are 50 nodes, whose node distribution corresponds to the characteristics of the scale-free network and the actual new epidemic regions.

### 4.2. Experimental analysis

Due to the scale-free social network constructed in the simulation, there is more than one node in the new outbreak area. Therefore, we will discuss the proposed method in the whole network with epidemics at one and two nodes respectively. At the same time, two sets of correlation strength data are used for experimental comparison and analysis, which makes the simulation experiment more effective.

- **Experiment 1**

Experiment 1 focuses on Group 1 data and discusses the construction of a regional outbreak risk transmission network when an outbreak occurs at one node and two nodes in a scale-free network, and the risk level of the whole region is classified using the proposed risk level classification method.

*Case 1: An outbreak has occurred in a region*.

**Figure 2:**
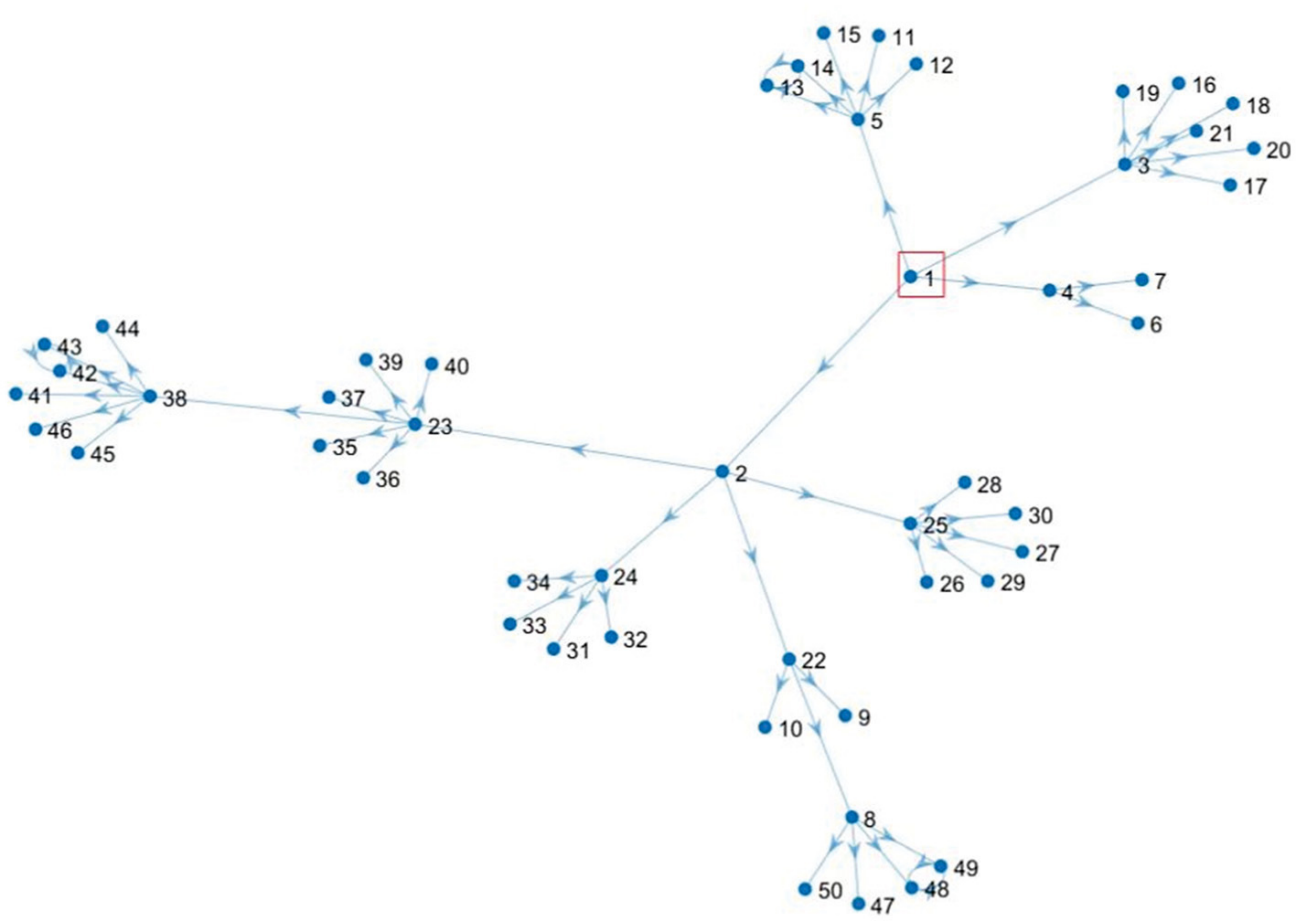
Directed scale-free social network during *v*_1_ outbreak.

Assuming the epidemic occurs in *v*_1_, the risk value of the *v*_1_ node is 1, and the resulting directed scale-free network is shown in Figure 2.

**Figure 3:**
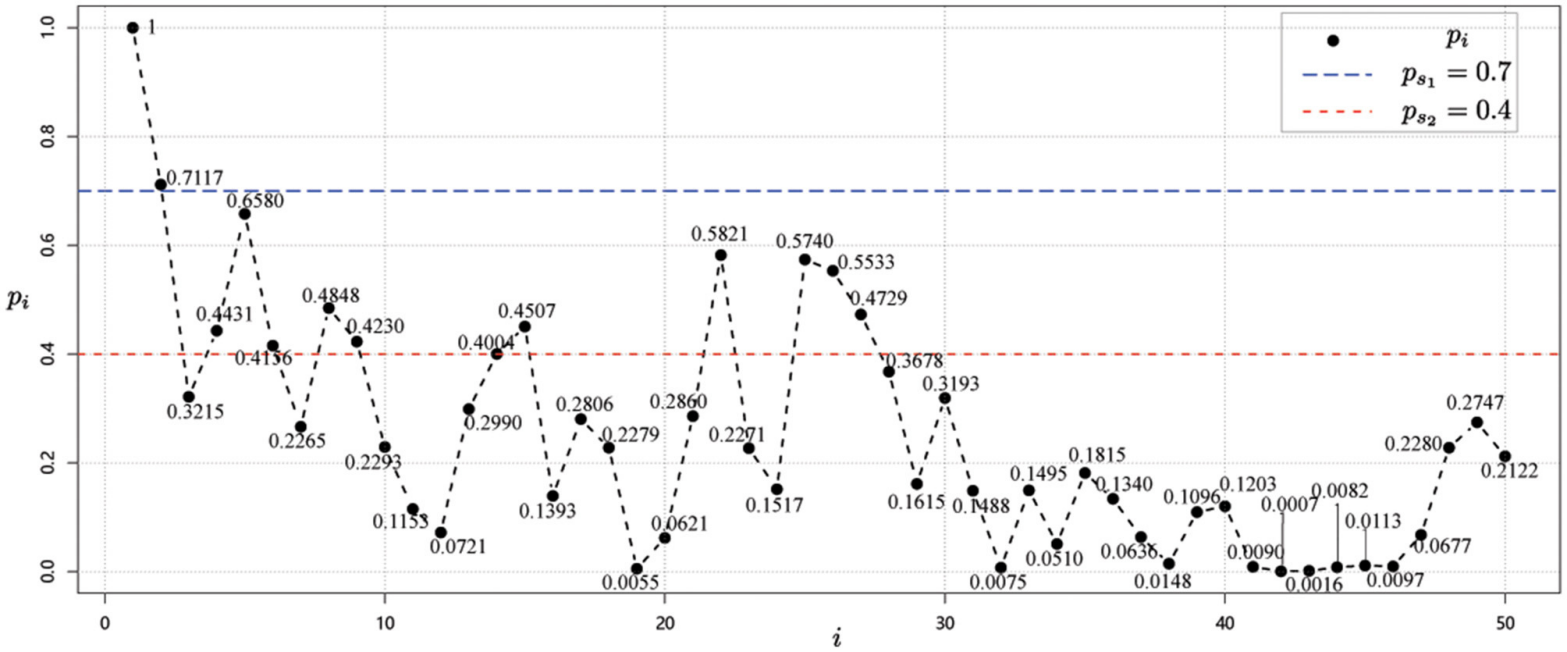
Risk value of each node *i, i* = 1, 2, ⋯ , 50 for data 1 for case 1.

Next, starting from the node *v*_1_ whose risk value is 1, and using the correlation strength in Table 2, we can calculate the risk values for each node *i, i* = 1, 2, ⋯ , 50 as shown in Figure 3.

Using the method that provided in Equations (3) and (4), the risk classification values are *p*_*s*_1__ = 0.7 and *p*_*s*_2__ = 0.4. Then, we can then obtain the risk level classification of each node from the risk values and the risk class classification criterion (37). As shown in Figure 3, it is easy to see that the risk level classification of each node is as given in Table 4.

**Table 4:** The risk grade classification of each node for data 1 for case 1.

**Risk levels**	**High-risk**	**Medium-risk**	**Low-risk**
Region	*v*_1_, *v*_2_	*v*_4_, *v*_5_, *v*_6_,	*v*_3_, *v*_7_, *v*_10_, *v*_11_, *v*_12_, *v*_13_,
		*v*_8_, *v*_9_, *v*_14_,	*v*_16_, *v*_17_, *v*_18_, *v*_19_, *v*_20_, *v*_21_,
		*v*_15_, *v*_22_, *v*_25_,	*v*_23_, *v*_24_, *v*_28_, *v*_29_, *v*_30_, *v*_31_,
		*v*_26_, *v*_27_	*v*_32_, *v*_33_, *v*_34_, *v*_35_, *v*_36_, *v*_37_,
			*v*_38_, *v*_39_, *v*_40_, *v*_41_, *v*_42_, *v*_43_,
			*v*_44_, *v*_45_, *v*_46_, *v*_47_, *v*_48_, *v*_49_, *v*_50_

*Case 2: Outbreaks occurred in two regions*.

In the case 2, we focus on the data of Group 1 and discuss the construction of a regional epidemic risk transmission network when an epidemic occurs at two nodes in a scale-free network, and the risk level of the whole region is classified using the proposed risk level classification method.

**Figure 4:**
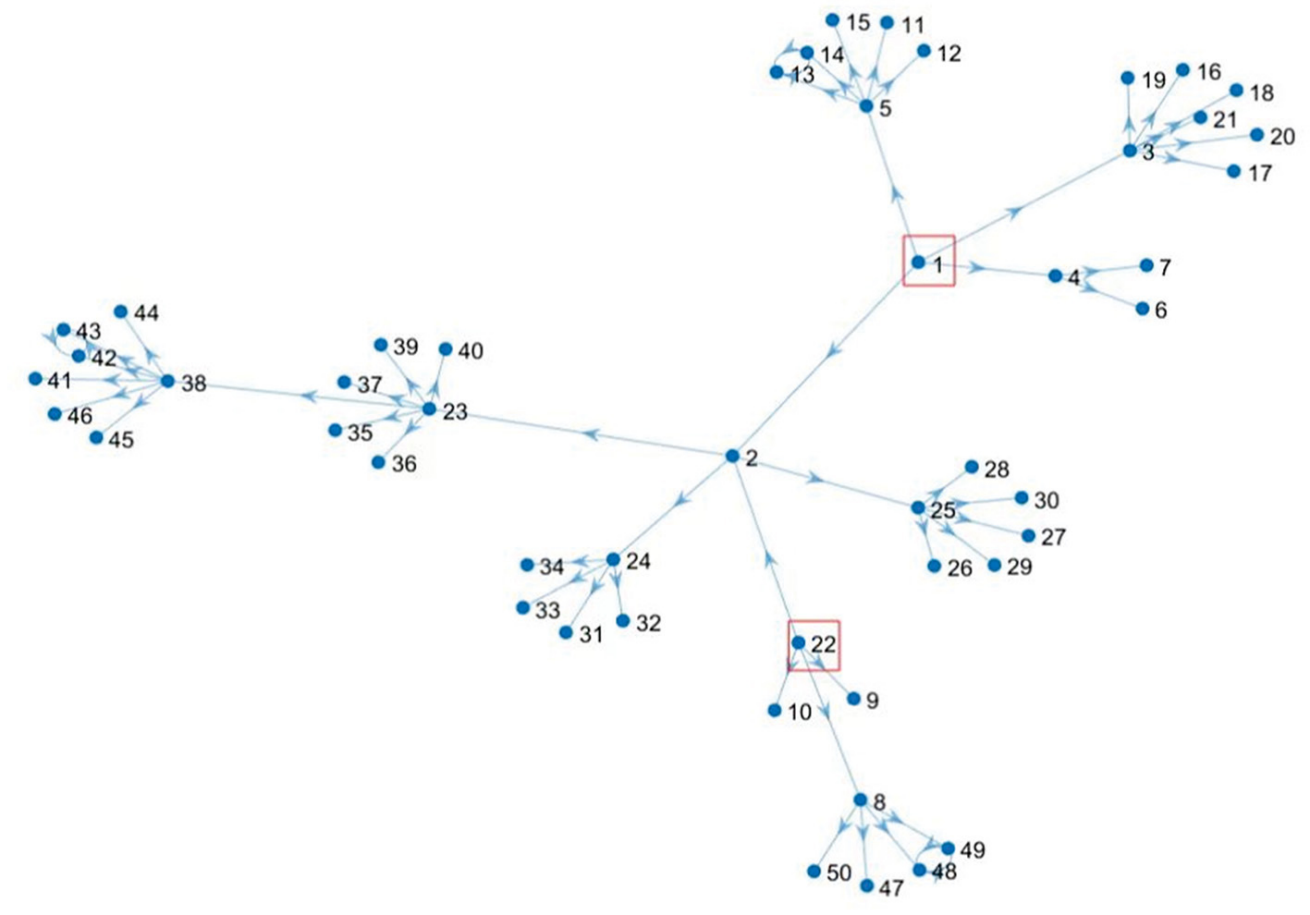
Directed scale-free social network during *v*_1_ and *v*_22_ outbreaks.

**Figure 5:**
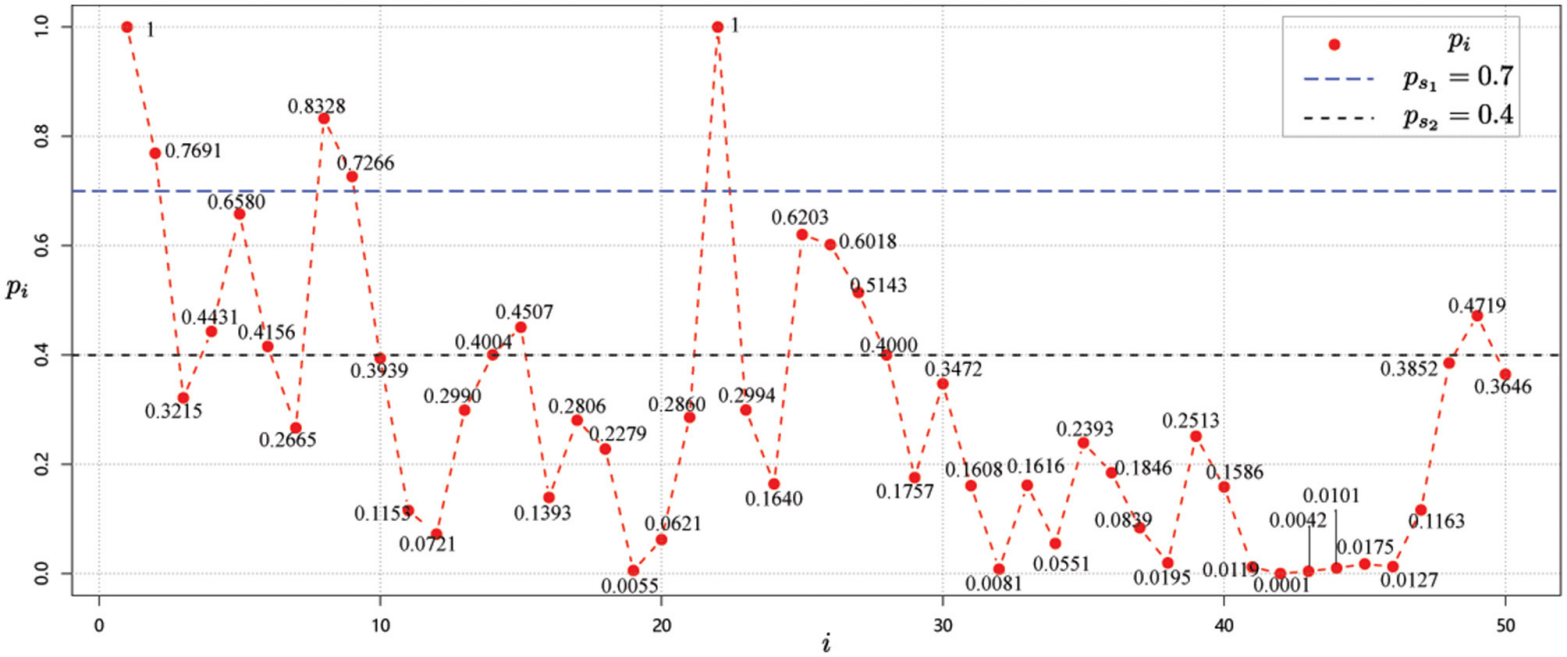
Risk value of each node *i, i* = 1, 2, ⋯ , 50 for data 1 for case 2.

Suppose the epidemic occurs at node *v*_1_ and node *v*_22_, then the risk value of node *v*_1_ and node *v*_22_ is 1, and the formed directed scale-free network is shown in Figure 4. Starting from node *v*_1_ and node *v*_22_ whose risk value is 1, we calculate the risk value of other nodes by considering the correlation strength in data 1, which is shown in Figure 5 below.

Then, according to the calculated risk values, we have the risk grade classification of each node, which is shown in Table 5 below.

**Table 5:** The risk grade classification of each node for data 1 for case 2.

**Risk levels**	**High-risk**	**Medium-risk**	**Low-risk**
Region	*v*_1_, *v*_2_,	*v*_4_, *v*_5_, *v*_6_, *v*_14_,	*v*_3_, *v*_7_, *v*_10_, *v*_11_, *v*_12_, *v*_13_,
	*v*_8_, *v*_9_,	*v*_15_, *v*_25_, *v*_26_,	*v*_16_, *v*_17_, *v*_18_, *v*_19_, *v*_20_,
	*v*_22_	*v*_27_, *v*_28_, *v*_49_	*v*_21_, *v*_23_, *v*_24_, *v*_29_, *v*_30_,
			*v*_31_, *v*_32_, *v*_33_, *v*_34_, *v*_35_,
			*v*_36_, *v*_37_, *v*_38_, *v*_39_, *v*_40_,
			*v*_41_, *v*_42_, *v*_43_, *v*_44_, *v*_45_,
			*v*_46_, *v*_47_, *v*_48_, *v*_50_

- **Experiment 2**

In Experiment 2, we consider the same method as in Experiment 1. For the data in Group 2, the classification of the regional risk level in the constructed scale-free social network is considered for the cases where the outbreak occurs in one node and in two nodes, respectively.

*Case 1: An outbreak has occurred in one region*.

**Figure 6:**
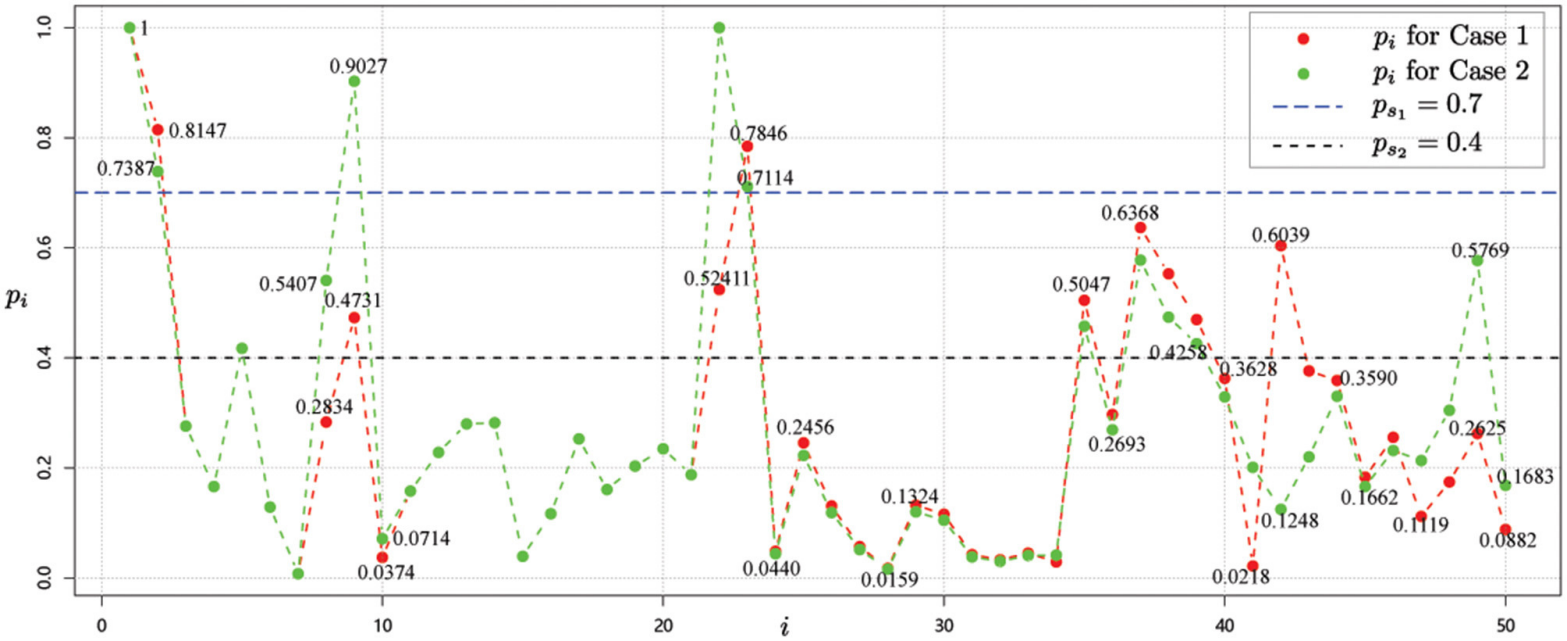
Risk value of each node *i, i* = 1, 2, ⋯ , 50 for data 2 for case 1 and case 2.

Now, considering the scale-free network as shown in Figure 2 for case 1. Starting from the node *v*_1_ with risk value 1, we get the risk values of other nodes by combining the association strength calculation in Group 2. Then, we get the risk value of each node *i, i* = 1, 2, ⋯ , 50 as shown in Figure 6.

Thus, based on the risk values obtained from the red dots in Figure 6 and the risk level classification guidelines in Table 1, we obtain the following risk level classification for each node.

- **High-risk regions:** *v*_1_, *v*_2_, *v*_22_, *v*_23_;
- **Medium-risk regions:** *v*_5_, *v*_9_, *v*_37_, *v*_38_, *v*_39_, *v*_42_, *v*_49_;
- **Low-risk regions:** *v*_3_, *v*_4_, *v*_6_, *v*_7_, *v*_8_, *v*_10_, *v*_11_, *v*_12_, *v*_13_, *v*_14_, *v*_15_, *v*_16_, *v*_17_, *v*_18_, *v*_19_, *v*_20_, *v*_21_, *v*_24_, *v*_25_, *v*_26_, *v*_27_, *v*_28_, *v*_29_, *v*_30_, *v*_31_, *v*_32_,*v*_33_, *v*_34_, *v*_35_, *v*_36_, *v*_40_, *v*_41_, *v*_43_, *v*_44_, *v*_45_, *v*_46_, *v*_47_, *v*_48_, *v*_50_.

*Case 2: Outbreaks occurred in two regions*

The situation is the same as shown in Figure 4. Next, starting with node *v*_1_ and node *v*_22_, whose risk value is 1, the risk value of other nodes is calculated for the correlation strength in Group 2, as shown by the green dots in Figure 6.

By considering the risk level classification guidelines in Table 1, we have

- **High-risk regions:** *v*_1_, *v*_2_, *v*_9_, *v*_22_, *v*_23_;
- **Medium-risk regions:** *v*_5_, *v*_8_, *v*_35_, *v*_37_, *v*_38_, *v*_39_, *v*_42_, *v*_49_;
- **Low-risk regions:** *v*_3_, *v*_4_, *v*_6_, *v*_7_, *v*_10_, *v*_11_, *v*_12_, *v*_13_, *v*_14_, *v*_15_, *v*_16_, *v*_17_, *v*_18_, *v*_19_, *v*_20_, *v*_21_, *v*_24_, *v*_25_, *v*_26_, *v*_27_, *v*_28_, *v*_29_, *v*_30_, *v*_31_, *v*_32_, *v*_33_, *v*_34_, *v*_36_, *v*_40_, *v*_41_, *v*_43_, *v*_44_, *v*_45_, *v*_46_, *v*_47_, *v*_48_,*v*_50_.

## 5. Conclusion

This paper uses graph theory and the risk assessment method to construct the epidemic risk classification method. In addition, the rationality and effectiveness of the classification method are verified by simulation. Furthermore, we use the MATLAB software to construct a scale-free network and generate the original the original data of the five required indicators. Then the risk classification degree in each node for two cases of the epidemic occurring in one node and two nodes are discussed. The experiment shows that the number of medium and high risk nodes does not show a significant increasing trend, and the number of high risk regions is relatively small compared to the number of medium risk regions, and the number of low risk regions is the largest, which is consistent with the classification of regional risk levels in the real society.

The construction of the social network of risk classification in new epidemic regions by the directed weighted scale-free network is more suitable for the transmission law of epidemic occurrence risk. It describes the transmission status of epidemic occurrence risk in social network relations. Furthermore, the established regional risk classification method can well classify the risk levels of different regions in the new epidemic area by determining the correlation function between the two regions and the risk value of the regional node. The experiment verified the rationality of the method, and it can provide a theoretical basis for the government to quickly judge the risk levels of different regions in epidemic prevention.

## Data availability statement

The datasets presented in this study can be found in online repositories. The names of the repository/repositories and accession number(s) can be found in the article/Supplementary material.

## Author contributions

XW: formal analysis and writing—review and editing. YL: methodology. CZ: resources. All authors contributed to the article and approved the submitted version.
