# Supplementary material for: Study on the regional risk classification method for the prevention and control of emerging infectious diseases based on directed graph theory
Source: Front Public Health. 2023 Sep 25;11:1211291. doi: 10.3389/fpubh.2023.1211291 (PMC10561095; doi:10.3389/fpubh.2023.1211291)
Supplement: Supplementary file 1 [file Data_Sheet_1.PDF]

Supplementary material for original data

In the event of an epidemic, appropriate management measures will be taken for high, medium, and low risk areas, and treating the community as a unit can facilitate prevention and control of the epidemic, reduce the chance of virus transmission, maximize normal life for more people, and reduce the impact of the epidemic on society.

In this paper, the raw data for the five indicators are constructed on a cell-by-cell basis as follows.

Original Data 1

Indicator 1: original data (*m*) for the distance (b_1)

$b_{1(1,2)} = 5492$	$b_{1(1,3)} = 24890$	$b_{1(1,4)} = 3419$	$b_{1(1,5)} = 5078$	
$b_{1(2,22)} = 6312$	$b_{1(2,23)} = 3004$	$b_{1(2,24)} = 16452$	$b_{1(2,25)} = 6624$	
$b_{1(3,16)} = 3344$	$b_{1(3,17)} = 6865$	$b_{1(3,18)} = 6865$	$b_{1(3,19)} = 1319565$	$b_{1(3,20)} = 14909$
$b_{1(3,21)} = 6866$				
$b_{1(4,6)} = 7238$	$b_{1(4,7)} = 4641$			
$b_{1(5,11)} = 13520$	$b_{1(5,12)} = 8458$	$b_{1(5,13)} = 5735$	$b_{1(5,14)} = 18551$	$b_{1(5,15)} = 5285$
$b_{1(8,22)} = 6427$	$b_{1(8,47)} = 10780$	$b_{1(8,48)} = 6093$	$b_{1(8,49)} = 14909$	$b_{1(8,50)} = 3378$
$b_{1(9,22)} = 5607$				
$b_{1(10,22)} = 3040$				
$b_{1(13,14)} = 7291$				
$b_{1(23,35)} = 6167$	$b_{1(23,36)} = 4757$	$b_{1(23,37)} = 21622$	$b_{1(23,38)} = 5031$	$b_{1(23,39)} = 6477$
$b_{1(23,40)} = 4088$				
$b_{1(24,31)} = 7567$	$b_{1(24,32)} = 3828$	$b_{1(24,33)} = 3828$	$b_{1(24,34)} = 25921$	
$b_{1(25,26)} = 7486$	$b_{1(25,27)} = 7486$	$b_{1(25,28)} = 5007$	$b_{1(25,29)} = 21854$	$b_{1(25,30)} = 4319$
$b_{1(38,41)} = 4698$	$b_{1(38,42)} = 331820$	$b_{1(38,43)} = 16606$	$b_{1(38,44)} = 3950$	$b_{1(38,45)} = 6917$
$b_{1(38,46)} = 5043$				
$b_{1(42,43)} = 19577$				
$b_{1(48,49)} = 6390$				

Indicator 2: Original data (persons/day) for personnel flow (b_2)

$b_{2(1,2)} = 4907$	$b_{2(1,3)} = 22165$	$b_{2(1,4)} = 3055$	$b_{2(1,5)} = 4536$	
$b_{2(2,22)} = 5629$	$b_{2(2,23)} = 2684$	$b_{2(2,24)} = 14699$	$b_{2(2,25)} = 5560$	
$b_{2(3,16)} = 2987$	$b_{2(3,17)} = 6133$	$b_{2(3,18)} = 6133$	$b_{2(3,19)} = 1178924$	$b_{2(3,20)} = 13320$
$b_{2(3,21)} = 6134$				
$b_{2(4,6)} = 6497$	$b_{2(4,7)} = 4146$			
$b_{2(5,11)} = 12079$	$b_{2(5,12)} = 7556$	$b_{1(5,13)} = 5735$	$b_{2(5,14)} = 16574$	$b_{2(5,15)} = 4722$
$b_{2(8,22)} = 5742$	$b_{2(8,47)} = 9631$	$b_{2(8,48)} = 5444$	$b_{2(8,49)} = 13320$	$b_{2(8,50)} = 3018$
$b_{2(9,22)} = 5009$				

$b_{2(10,22)} = 2716$				
$b_{2(13,14)} = 6514$				
$b_{2(23,35)} = 5510$	$b_{2(23,36)} = 4250$	$b_{2(23,37)} = 19318$	$b_{2(23,38)} = 4495$	$b_{2(23,39)} = 5786$
$b_{2(23,40)} = 3652$				
$b_{2(24,31)} = 6761$	$b_{2(24,32)} = 3420$	$b_{2(24,33)} = 3420$	$b_{2(24,34)} = 23158$	
$b_{2(25,26)} = 6688$	$b_{2(25,27)} = 6688$	$b_{2(25,28)} = 4473$	$b_{2(25,29)} = 19525$	$b_{2(25,30)} = 3856$
$b_{2(38,41)} = 4197$	$b_{2(38,42)} = 296455$	$b_{2(38,43)} = 14837$	$b_{2(38,44)} = 3529$	$b_{2(38,45)} = 6180$
$b_{2(38,46)} = 4505$				
$b_{2(42,43)} = 17491$				
$b_{2(48,49)} = 5709$				

Indicator 3: Original data (CNY) for economic tranffic (b_3)

$b_{3(1,2)} = 7253$	$b_{3(1,3)} = 32766$	$b_{3(1,4)} = 4516$	$b_{3(1,5)} = 6706$	
$b_{3(2,22)} = 8336$	$b_{3(2,23)} = 3968$	$b_{3(2,24)} = 21728$	$b_{3(2,25)} = 8219$	
$b_{3(3,16)} = 4416$	$b_{3(3,17)} = 9066$	$b_{3(3,18)} = 9066$	$b_{3(3,19)} = 1742757$	$b_{3(3,20)} = 19690$
$b_{3(3,21)} = 9067$				
$b_{3(4,6)} = 9560$	$b_{3(4,7)} = 6129$			
$b_{3(5,11)} = 17856$	$b_{3(5,12)} = 11170$	$b_{3(5,13)} = 7574$	$b_{3(5,14)} = 24501$	$b_{3(5,15)} = 6980$
$b_{3(8,22)} = 8488$	$b_{3(8,47)} = 14238$	$b_{3(8,48)} = 8047$	$b_{3(8,49)} = 19690$	$b_{3(8,50)} = 4462$
$b_{3(9,22)} = 7405$				
$b_{3(10,22)} = 4014$				
$b_{3(13,14)} = 9629$				
$b_{3(23,35)} = 6556$	$b_{3(23,36)} = 3832028$	$b_{3(23,37)} = 8271$	$b_{3(23,38)} = 6791$	$b_{3(23,39)} = 6100$
$b_{3(23,40)} = 4713$				
$b_{3(24,31)} = 8866$	$b_{3(24,32)} = 6965$	$b_{3(24,33)} = 9474$	$b_{3(24,34)} = 9638$	
$b_{3(25,26)} = 5430$	$b_{3(25,27)} = 23604$	$b_{3(25,28)} = 7277$	$b_{3(25,29)} = 5494$	$b_{3(25,30)} = 4799$
$b_{3(38,41)} = 4324$	$b_{3(38,42)} = 27354$	$b_{3(38,43)} = 7175$	$b_{3(38,44)} = 7100$	$b_{3(38,45)} = 35742$
$b_{3(38,46)} = 4983$				
$b_{3(42,43)} = 5259$				
$b_{3(48,49)} = 7115$				

Indicator 4: Original data for transportation convenience (b_4)

$b_{4(1,2)} = 12833$	$b_{4(1,3)} = 57970$	$b_{4(1,4)} = 7990$	$b_{4(1,5)} = 11865$	
$b_{4(2,22)} = 14748$	$b_{4(2,23)} = 7020$	$b_{4(2,24)} = 38433$	$b_{4(2,25)} = 14542$	
$b_{4(3,16)} = 7813$	$b_{4(3,17)} = 16041$	$b_{4(3,18)} = 16041$	$b_{4(3,19)} = 3083340$	$b_{4(3,20)} = 34836$
$b_{4(3,21)} = 16042$				
$b_{4(4,6)} = 16913$	$b_{4(4,7)} = 10844$			
$b_{4(5,11)} = 31591$	$b_{4(5,12)} = 19762$	$b_{4(5,13)} = 13401$	$b_{4(5,14)} = 43347$	$b_{4(5,15)} = 12350$

$b_{4(8,22)}=15016$	$b_{4(8,47)}=25190$	$b_{4(8,48)}=14237$	$b_{4(8,49)}=34836$	$b_{4(8,50)}=7894$
$b_{4(9,22)}=13101$				
$b_{4(10,22)}=7103$				
$b_{4(13,14)}=17036$				
$b_{4(23,35)}=14411$	$b_{4(23,36)}=11114$	$b_{4(23,37)}=50523$	$b_{4(23,38)}=11756$	$b_{4(23,39)}=15134$
$b_{4(23,40)}=9551$				
$b_{4(24,31)}=17681$	$b_{4(24,32)}=8943$	$b_{4(24,33)}=8943$	$b_{4(24,34)}=60567$	
$b_{4(25,26)}=17492$	$b_{4(25,27)}=17492$	$b_{4(25,28)}=11699$	$b_{4(25,29)}=51064$	$b_{4(25,30)}=10092$
$b_{4(38,41)}=10977$	$b_{4(38,42)}=775343$	$b_{4(38,43)}=38803$	$b_{4(38,44)}=9230$	$b_{4(38,45)}=16163$
$b_{4(38,46)}=11783$				
$b_{4(42,43)}=45745$				
$b_{4(48,49)}=14932$				

Indicator 5: Original data (pcs/day) for logistics intensity (b_5)

$b_{5(1,2)}=18536$	$b_{5(1,3)}=83735$	$b_{5(1,4)}=11541$	$b_{5(1,5)}=17138$	
$b_{5(2,22)}=21302$	$b_{5(2,23)}=10139$	$b_{5(2,24)}=55528$	$b_{5(2,25)}=21005$	
$b_{5(3,16)}=11285$	$b_{5(3,17)}=23170$	$b_{5(3,18)}=23170$	$b_{5(3,19)}=4453713$	$b_{5(3,20)}=50319$
$b_{5(3,21)}=23172$				
$b_{5(4,6)}=24330$	$b_{5(4,7)}=15664$			
$b_{5(5,11)}=45631$	$b_{5(5,12)}=28545$	$b_{5(5,13)}=19357$	$b_{5(5,14)}=62612$	$b_{5(5,15)}=17838$
$b_{5(8,22)}=21690$	$b_{5(8,47)}=36385$	$b_{5(8,48)}=20565$	$b_{5(8,49)}=50319$	$b_{5(8,50)}=11403$
$b_{5(9,22)}=18924$				
$b_{5(10,22)}=10259$				
$b_{5(13,14)}=24607$				
$b_{5(23,35)}=20815$	$b_{5(23,36)}=16054$	$b_{5(23,37)}=72978$	$b_{5(23,38)}=16981$	$b_{5(23,39)}=21860$
$b_{5(23,40)}=3796$				
$b_{5(24,31)}=15540$	$b_{5(24,32)}=12918$	$b_{5(24,33)}=12918$	$b_{5(24,34)}=87486$	
$b_{5(25,26)}=25266$	$b_{5(25,27)}=25266$	$b_{5(25,28)}=16898$	$b_{5(25,29)}=73760$	$b_{5(25,30)}=14577$
$b_{5(38,41)}=15856$	$b_{5(38,42)}=1119940$	$b_{5(38,43)}=56049$	$b_{5(38,44)}=13332$	$b_{5(38,45)}=23347$
$b_{5(38,46)}=17020$				
$b_{5(42,43)}=66076$				
$b_{5(48,49)}=21568$				

Original Data 2

Indicator 1: original data (*m*) for the distance (b_1)

$b_{1(1,2)} = 6287$	$b_{1(1,3)} = 21298$	$b_{1(1,4)} = 12517$	$b_{1(1,5)} = 3220$	
$b_{1(2,22)} = 4964$	$b_{1(2,23)} = 7224$	$b_{1(2,24)} = 4599$	$b_{1(2,25)} = 23266$	
$b_{1(3,16)} = 3317$	$b_{1(3,17)} = 7069$	$b_{1(3,18)} = 4493$	$b_{1(3,19)} = 5682$	$b_{1(3,20)} = 6565$
$b_{1(3,21)} = 5245$				
$b_{1(4,6)} = 6129$	$b_{1(4,7)} = 3696$			
$b_{1(5,11)} = 29239$	$b_{1(5,12)} = 4220$	$b_{1(5,13)} = 5249$	$b_{1(5,14)} = 5410$	$b_{1(5,15)} = 7269$
$b_{1(8,22)} = 4172$	$b_{1(8,47)} = 3046$	$b_{1(8,48)} = 4326$	$b_{1(8,49)} = 7291$	$b_{1(8,50)} = 24015$
$b_{1(9,22)} = 6966$				
$b_{1(10,22)} = 5510$				
$b_{1(13,14)} = 7291$				
$b_{1(23,35)} = 4964$	$b_{1(23,36)} = 2901499$	$b_{1(23,37)} = 6263$	$b_{1(23,38)} = 5142$	$b_{1(23,39)} = 4618$
$b_{1(23,40)} = 3568$				
$b_{1(24,31)} = 6713$	$b_{1(24,32)} = 5274$	$b_{1(24,33)} = 7173$	$b_{1(24,34)} = 7298$	
$b_{1(25,26)} = 4111$	$b_{1(25,27)} = 17872$	$b_{1(25,28)} = 5510$	$b_{1(25,29)} = 4160$	$b_{1(25,30)} = 3634$
$b_{1(38,41)} = 3274$	$b_{1(38,42)} = 20712$	$b_{1(38,43)} = 5433$	$b_{1(38,44)} = 5376$	$b_{1(38,45)} = 27063$
$b_{1(38,46)} = 3773$				
$b_{1(42,43)} = 3982$				
$b_{1(48,49)} = 5387$				

Indicator 2: Original data (persons/day) for personnel flow (b_2)

$b_{2(1,2)} = 5617$	$b_{2(1,3)} = 19028$	$b_{2(1,4)} = 11183$	$b_{2(1,5)} = 2877$	
$b_{2(2,22)} = 4435$	$b_{2(2,23)} = 6454$	$b_{2(2,24)} = 4109$	$b_{2(2,25)} = 20786$	
$b_{2(3,16)} = 2964$	$b_{2(3,17)} = 6315$	$b_{2(3,18)} = 4014$	$b_{2(3,19)} = 5076$	$b_{2(3,20)} = 5865$
$b_{2(3,21)} = 4686$				
$b_{2(4,6)} = 5476$	$b_{2(4,7)} = 3302$			
$b_{2(5,11)} = 26122$	$b_{2(5,12)} = 3770$	$b_{1(5,13)} = 4689$	$b_{2(5,14)} = 4834$	$b_{2(5,15)} = 6494$
$b_{2(8,22)} = 3728$	$b_{2(8,47)} = 2721$	$b_{2(8,48)} = 3865$	$b_{2(8,49)} = 6514$	$b_{2(8,50)} = 21455$
$b_{2(9,22)} = 6223$				
$b_{2(10,22)} = 4923$				
$b_{2(13,14)} = 6514$				
$b_{2(23,35)} = 4435$	$b_{2(23,36)} = 2592254$	$b_{2(23,37)} = 5595$	$b_{2(23,38)} = 4594$	$b_{2(23,39)} = 4126$
$b_{2(23,40)} = 3188$				
$b_{2(24,31)} = 5997$	$b_{2(24,32)} = 4712$	$b_{2(24,33)} = 6409$	$b_{2(24,34)} = 6520$	
$b_{2(25,26)} = 3673$	$b_{2(25,27)} = 15967$	$b_{2(25,28)} = 4923$	$b_{2(25,29)} = 3717$	$b_{2(25,30)} = 3247$
$b_{2(38,41)} = 2925$	$b_{2(38,42)} = 18504$	$b_{2(38,43)} = 4854$	$b_{2(38,44)} = 4803$	$b_{2(38,45)} = 24178$

$b_{2(38,46)} = 3371$

$b_{2(42,43)} = 3557$				
$b_{2(48,49)} = 4813$				

Indicator 3: Original data (CNY) for economic traffic (b_3)

$b_{3(1,2)} = 8303$	$b_{3(1,3)} = 28129$	$b_{3(1,4)} = 16531$	$b_{3(1,5)} = 4253$	
$b_{3(2,22)} = 6556$	$b_{3(2,23)} = 9540$	$b_{3(2,24)} = 6074$	$b_{3(2,25)} = 30728$	
$b_{3(3,16)} = 4381$	$b_{3(3,17)} = 9335$	$b_{3(3,18)} = 5934$	$b_{3(3,19)} = 7504$	$b_{3(3,20)} = 8670$
$b_{3(3,21)} = 6927$				
$b_{3(4,6)} = 8095$	$b_{3(4,7)} = 4882$			
$b_{3(5,11)} = 38616$	$b_{3(5,12)} = 5573$	$b_{3(5,13)} = 6932$	$b_{3(5,14)} = 7145$	$b_{3(5,15)} = 9600$
$b_{3(8,22)} = 5511$	$b_{3(8,47)} = 4023$	$b_{3(8,48)} = 5713$	$b_{3(8,49)} = 9629$	$b_{3(8,50)} = 31716$
$b_{3(9,22)} = 9200$				
$b_{3(10,22)} = 7277$				
$b_{3(13,14)} = 9629$				
$b_{3(23,35)} = 6556$	$b_{3(23,36)} = 3832028$	$b_{3(23,37)} = 8271$	$b_{3(23,38)} = 6791$	$b_{3(23,39)} = 6100$
$b_{3(23,40)} = 4713$				
$b_{3(24,31)} = 8866$	$b_{3(24,32)} = 6965$	$b_{3(24,33)} = 9474$	$b_{3(24,34)} = 9638$	
$b_{3(25,26)} = 5430$	$b_{3(25,27)} = 23604$	$b_{3(25,28)} = 7277$	$b_{3(25,29)} = 5494$	$b_{3(25,30)} = 4799$
$b_{3(38,41)} = 4324$	$b_{3(38,42)} = 27354$	$b_{3(38,43)} = 7175$	$b_{3(38,44)} = 7100$	$b_{3(38,45)} = 35742$
$b_{3(38,46)} = 4983$				
$b_{3(42,43)} = 5259$				
$b_{3(48,49)} = 7115$				

Indicator 4: Original data for transportation convenience (b_4)

$b_{4(1,2)} = 14690$	$b_{4(1,3)} = 49766$	$b_{4(1,4)} = 29247$	$b_{4(1,5)} = 7524$	
$b_{4(2,22)} = 11599$	$b_{4(2,23)} = 16879$	$b_{4(2,24)} = 10747$	$b_{4(2,25)} = 54364$	
$b_{4(3,16)} = 7752$	$b_{4(3,17)} = 16517$	$b_{4(3,18)} = 10498$	$b_{4(3,19)} = 13276$	$b_{4(3,20)} = 15339$
$b_{4(3,21)} = 12256$				
$b_{4(4,6)} = 14322$	$b_{4(4,7)} = 8637$			
$b_{4(5,11)} = 68320$	$b_{4(5,12)} = 9859$	$b_{4(5,13)} = 12265$	$b_{4(5,14)} = 12642$	$b_{4(5,15)} = 16985$
$b_{4(8,22)} = 9749$	$b_{4(8,47)} = 7117$	$b_{4(8,48)} = 10108$	$b_{4(8,49)} = 17036$	$b_{4(8,50)} = 56113$
$b_{4(9,22)} = 16277$				
$b_{4(10,22)} = 12874$				
$b_{4(13,14)} = 17036$				
$b_{4(23,35)} = 11599$	$b_{4(23,36)} = 6779741$	$b_{4(23,37)} = 14634$	$b_{4(23,38)} = 12041$	$b_{4(23,39)} = 10792$
$b_{4(23,40)} = 8815$				

$b_{4(24,31)} = 15685$	$b_{4(24,32)} = 12323$	$b_{4(24,33)} = 16762$	$b_{4(24,34)} = 17052$	
$b_{4(25,26)} = 9607$	$b_{4(25,27)} = 41760$	$b_{4(25,28)} = 12874$	$b_{4(25,29)} = 9721$	$b_{4(25,30)} = 8491$
$b_{4(38,41)} = 7651$	$b_{4(38,42)} = 48396$	$b_{4(38,43)} = 12694$	$b_{4(38,44)} = 12562$	$b_{4(38,45)} = 63236$
$b_{4(38,46)} = 11783$				
$b_{4(42,43)} = 9304$				
$b_{4(48,49)} = 12588$				

Indicator 5: Original data (pcs/day) for logistics intensity (b_5)

$b_{5(1,2)} = 21219$	$b_{5(1,3)} = 71884$	$b_{5(1,4)} = 42245$	$b_{5(1,5)} = 10869$	
$b_{5(2,22)} = 16755$	$b_{5(2,23)} = 24381$	$b_{5(2,24)} = 15523$	$b_{5(2,25)} = 78526$	
$b_{5(3,16)} = 11197$	$b_{5(3,17)} = 23857$	$b_{5(3,18)} = 15163$	$b_{5(3,19)} = 19177$	$b_{5(3,20)} = 22157$
$b_{5(3,21)} = 17703$				
$b_{5(4,6)} = 20688$	$b_{5(4,7)} = 12476$			
$b_{5(5,11)} = 98685$	$b_{5(5,12)} = 14241$	$b_{5(5,13)} = 17716$	$b_{5(5,14)} = 18260$	$b_{5(5,15)} = 24534$
$b_{5(8,22)} = 14083$	$b_{5(8,47)} = 10280$	$b_{5(8,48)} = 14601$	$b_{5(8,49)} = 24607$	$b_{5(8,50)} = 81052$
$b_{5(9,22)} = 23511$				
$b_{5(10,22)} = 18596$				
$b_{5(13,14)} = 24607$				
$b_{5(23,35)} = 20815$	$b_{5(23,36)} = 16054$	$b_{5(23,37)} = 72978$	$b_{5(23,38)} = 16981$	$b_{5(23,39)} = 21860$
$b_{5(23,40)} = 3796$				
$b_{5(23,35)} = 16755$	$b_{5(23,36)} = 9792960$	$b_{5(23,37)} = 21138$	$b_{5(23,38)} = 17354$	$b_{5(23,39)} = 15588$
$b_{5(23,40)} = 12043$				
$b_{5(24,31)} = 22657$	$b_{5(24,32)} = 17799$	$b_{5(24,33)} = 24212$	$b_{5(24,34)} = 24631$	
$b_{5(25,26)} = 13877$	$b_{5(25,27)} = 60302$	$b_{5(25,28)} = 18596$	$b_{5(25,29)} = 14041$	$b_{5(25,30)} = 12265$
$b_{5(38,41)} = 11051$	$b_{5(38,42)} = 69905$	$b_{5(38,43)} = 18336$	$b_{5(38,44)} = 18146$	$b_{5(38,45)} = 91340$
$b_{5(38,46)} = 12733$				
